# Embracing challenging complexity: exploring handwashing behavior from a combined socioecological and intersectional perspective in Sierra Leone

**DOI:** 10.1186/s12889-021-11923-1

**Published:** 2021-10-14

**Authors:** Hanna Luetke Lanfer, Doreen Reifegerste

**Affiliations:** School of Public Health, Bielefeld University, Universitaetsstrasse 25, 33615 Bielefeld, Germany

**Keywords:** Health inequalities, Socioecological model, Intersectionality, Handwashing, Qualitative method, Social determinants of health, Low-income countries

## Abstract

**Background:**

Handwashing with soap is a cost-effective, efficient health behavior to prevent various diseases. Despite its immense health benefits, the lowest prevalence of handwashing is found in low-income countries. Here, its practice is not only determined by individual behavior, but also heavily shaped by deprivations in the social and structural ecology. Moreover, handwashing barriers are not equally experienced as overlapping social identities (e.g., age and gender) intersect and create inequities between members of different social groups. To embrace the complexities of handwashing beyond individual-level behavior and singular social identities, a combined socioecological and intersectional perspective is employed. This multi-level approach with regards to intersecting privileges and disadvantages serves as a basis to promote this highly important health behavior.

**Methods:**

This study used a qualitative, theory-based approach and combined data from two samples: experts in health promotion (*n* = 22) and local citizens stratified by gender and rural/urban location (*n* = 56). Data was collected in face-to-face interviews in Sierra Leone between November 2018 and January 2019 and analyzed using thematic analysis and typology of the qualitative data.

**Results:**

The conceptualization of multi-level determinants of handwashing within a socioecological model showed the high relevance of inhibiting social and structural factors for handwashing practice. By establishing seven distinguishing social identity dimensions, data demonstrates that individuals within the same social setting yet with distinct social identities experience strikingly differing degrees of power and privileges to enact handwashing. While a local leader is influential and may also change structural-level determinants, a young, rural wife experiences multiple social and structural constraints to perform handwashing with soap, even if she has high handwashing intentions.

**Conclusion:**

This study provides a holistic analytical framework for the identification of determinants on multiple levels and accumulating intersections of socially produced inequalities for handwashing and is applicable to other health topics. As the exploration of handwashing was approached from a solution-focused instead of a problem-focused perspective, the analysis can guide multi-level intervention approaches (e.g., using low-cost, participatory activities at the community level to make use of the available social capital).

**Supplementary Information:**

The online version contains supplementary material available at 10.1186/s12889-021-11923-1.

## Background

From a public health viewpoint, handwashing with soap is one of the most cost-effective means to prevent a variety of infectious diseases and improve the health outcomes of adults and children alike [1–3]. While the current COVID-19 pandemic has increased handwashing practice across the world [4], current and previous studies have pointed to the lowest prevalence of handwashing with soap in Sub-Saharan Africa which is also the region with the highest number of low-income countries and generally poor population health [4–6]. Researchers agree that health-related behaviors such as handwashing with soap are not only determined by individual behavior but heavily shaped by a multitude of contextual factors [7, 8]. Studies have shown that the available resources [7], place of residence [8], gender [9], social status [10] and education [11] can influence whether a person practices handwashing or not. Moreover, it has been shown that barriers to handwashing are not experienced equally as overlapping social identity dimensions intersect and distinctly shape privileges and disadvantages associated with handwashing practice [9]. This is especially true in low-income countries (LIC), where various forms of inequalities overlap and exacerbate its barriers, requiring more user commitment, effort and time to sustain handwashing practice than from people living in high-income countries [12, 13].

To increase handwashing and other hygiene behaviors in deprived settings with poor health outcomes, several frameworks have been developed over the years (e.g., the Integrated Behaviour Model for Water, Sanitation and Hygiene [14] or the FOAM framework, acronym for Focus, Opportunity, Ability and Motivation [15]). In their systematic review of handwashing determinants, White and colleagues [16] have reviewed these frameworks and the determinants related to handwashing and behavior change. While the authors describe great overlaps between the determinants of different frameworks, they also conclude that there are major gaps in the literature on handwashing determinants:

“Our review indicates that the overall quality of the evidence on this topic remains poor and that the literature is skewed towards reporting certain types of determinants (e.g. characteristic traits, infrastructure and executive brain functions such as knowledge, risk and discounts) at the expense of a more complete understanding of what drives HWWS [handwashing with water and soap]” ([16], p. 11)

This bias may result in overlooking the complexity of context factors, especially in LICs with multiple barriers to handwashing and differences between more and less privileged members of social groups in a shared setting. Consequently, this study aims to identify what and who influences decisions about handwashing in a severely deprived setting by employing a combined socioecological and intersectional framework to promote hand hygiene.

### Socioecological models

A frequently cited framework that allows for the identification of contextual factors of human behavior are socioecological models (SEMs). SEMs use the analogy of the natural ecosystem and posit that individuals are embedded in various interdependent subsystems [17]. Since their initial constitution in the seventies, SEMs have been conceived as a framework that can be adapted and changed to suit research and interventions in different disciplines, including public health research. Here, socioecological approaches are used to explore potential relationships between risk factors, which might be situated in the individual as well as in the contextual environment, and health disparities in certain population groups [18, 19]. In contrast to psychological models of health behavior which focus mainly on individual behavior, SEMs take into account not only immediate external influences such as peer pressure from interpersonal relationships but also peripheral factors such as the community, governing entities and the infrastructure and how these influences interact with people’s individual beliefs, behavioral intentions and enactments of behavior [20]. While SEMs can be used to explore and conceptualize health behaviors in their specific context, they can also serve to inform multi-level interventions [20, 21] (e.g., promoting HIV services for pregnant women in Kenya [22] or safe food handling in South Africa [23]). Thus, to identify and disentangle interrelated behavioral and contextual factors of handwashing and inform intervention development, SEMs provide a suitable and established theoretical framework.

However, so far only a few studies analyzed domestic handwashing on the basis of SEMs. In a study in Malawi, an SEM was used to develop a handwashing program for schools [24]. The researchers found that handwashing was not supported at the community level, which resulted in reluctance among the students to wash their hands and regular theft of handwashing supplies as soon as new equipment was bought. In addition, barrier analyses from various countries indicate that the perceived affordability of the relevant materials and resources (e.g., a handwashing station, soap and water [25]), a lack of trust in the biomedical health system and its messengers [26] and low health literacy [27] are relevant determinants of handwashing behavior. As existing studies demonstrate that handwashing with soap is shaped by determinants on multiple levels, but mostly lack an overall SEM perspective, the first research question is:

RQ 1: What are relevant determinants of handwashing on the individual, social and structural level in Sierra Leone?

### Intersectionality

While an SEM approach allows for the structured exploration of determinants on different levels and their influence on individual beliefs and perceptions, research indicates that barriers are not experienced equally within a given environment. Instead, in a shared social setting, different dimensions of a person’s social identity intersect and distinctly shape their experiences. This observation was first conceptualized in the intersectionality framework by Black feminists in the nineties. Kimberlé Crenshaw [28] argued that violence and discrimination against women were not only determined by their gender, but also by other dimensions of their identity, e.g., their ethnicity, social class, age, educational level and socioeconomic status (SES). In one of her first publications on intersectionality, Crenshaw [28] demonstrated the case of Black women whose experiences of sexism and racism did not equal those of White women or Black men, but whose multiplied inequalities and marginalization remained nonetheless obscured in public discourses. Different from approaches that center on singular, sometimes added categories of difference and separate foci of research, e.g., gender or SES, intersectionality recognizes the “wider social processes through which multiple, intersecting inequalities are reproduced and perpetuated” ([29], p. 46).

Since its introduction, the intersectional framework has influenced research in various fields (see Hill Collins & Bilge [30] for an overview), including its recognition as an analytical tool in health-related research to provide insights into the complex nature of health, power and inequalities [31–34]. In this, intersectionality provides a conceptual framework to understand distinct experiences of privilege and disadvantage alongside dimensions of social stratification and structural drivers that define and reinforce health inequalities [33, 35]. In LICs, often at least one dimension of a person’s social identity is deprivileged [29, 36]. Scholars have established that in LICs, hygiene behaviors can intersect with inequalities of poverty status [37], gender [38], a rural-urban divide [5], age [39], education [40], ethnicity [41] and social status [42]. Looking at these dimensions separately obscures their interconnectedness and the overlapping disadvantages experienced by some groups in a given social setting. In a study across 12 villages in two Indian states, Mitra and Rao [41] found that women’s hygiene behaviors intersected with their caste, household composition and employment status, resulting in different degrees of handwashing that depended on a woman’s compounding disadvantages. In another study in an area affected by frequent water shortages in Botswana, researchers found that unemployed mothers with a low educational status struggled to balance household duties with time pressure to acquire sufficient water and comply with social norms of hygiene [43]. In contrast, higher educated women, including mothers, in the same area were more likely to be employed and, thus, able to compensate for water shortages by buying water.

By blending the socioecological and intersectional approach, research can link the underlying individual, social and structural determinants of hygiene behaviors with intersecting dimensions of social inequalities. It allows for the nuanced characterization of different degrees of vulnerability within an overall deprived setting. While the idea of incorporating the two approaches for the exploration of complex problems is not new (e.g., Levine & Breshears [44] to investigate discrimination among people with disabilities; Standley [45] for suicide prevention; Brinkley-Rubenstein & Mann [46] for health disparities among minorities) its application remains scarce. To our knowledge, handwashing behavior has never been explored from a combined socioecological and intersectional perspective. Thus, to explore, analyze and address the intersecting social identity dimensions with other determinants of handwashing behavior, we ask:

RQ 2: Which social identity dimensions intersect with handwashing practice and other multi-level determinants in Sierra Leone?

To increase handwashing practice and design targeted interventions, it is important to identify different degrees of privilege and disadvantage and how social and structural determinants in a low-income setting shape handwashing uptake. Characterizing the most marginalized, disadvantaged social position is key to recognizing their barriers and to developing strategies to strengthen handwashing uptake. Likewise, characterizing the features and social dimensions of social positions who experience relative privileges in the same social setting can equally contribute to renegotiating power structures related to handwashing practice. Thus, we ask:

RQ 3: Who are the most and least disadvantaged social positions to practice handwashing in Sierra Leone?

## Methods

### Study design and setting

This research is part of a larger study to promote handwashing in a low-income environment [XXX, anonymized for peer review]. For an in-depth exploration of multi-level, intersecting barriers and enablers of handwashing practice in a specific cultural and socioeconomic context, a qualitative approach was chosen. This allows exploring the specific social norms connected with handwashing that would not be visible or explainable within a standardized questionnaire. The present study applied a theory-based approach, where prior defined research questions guided data collection and analysis. To enhance its quality and provide multiple perspectives on handwashing practice, the study combines semi-structured interviews from two different samples (*n* = 22 experts in health promotion and health policymaking; and *n* = 56 local citizens stratified by gender and rural/urban location).

Sierra Leone was chosen as the country of research for this study. According to the Human Development Index [47], 57.9% of the total population in Sierra Leone is considered multidimensionally poor and another 19.6% is vulnerable to multidimensional poverty. In addition, less than one third of the adult population (32.4%) is literate [48]. Thus, by investigating handwashing behavior among groups with a low SES, our study focuses on a substantial proportion of the total population in the context of Sierra Leone. At the same time, this is the group that is most relevant for changing handwashing behavior due to a high prevalence of nutritional deficiencies, pneumonia, diarrhea and other infectious diseases which coincide with a low presence of functioning handwashing stations equipped with soap and water (estimated at 15% in rural and 33% in urban areas) and an assumed low performance of hygiene behaviors [49].

Field work for this study was conducted between November 2018 and January 2019 in six different districts of Sierra Leone. Interviews with local citizens were all conducted in Krio, one of the most commonly spoken local languages; expert interviews mainly took place in English, except for two interviewees who preferred to answer in their native languages Krio and German. The research team consisted of the first author and two local research assistants (one woman, one man) who were trained extensively beforehand. All members of the field research team spoke both Krio and English fluently.

### Participants and procedure

Purposeful and snowball sampling was conducted to recruit participants for the two samples of this study.

#### Experts

To identify interviewees who were experienced in developing and/or disseminating health messages on hygiene behaviors, we relied on a network of health professionals established during the West African Ebola outbreak and identified authors of relevant health promotion publications. These people were contacted by email or through their organization. Moreover, these interviewees were asked for further referrals. Data saturation determined the end of data collection, as the sample was heterogenous. Only one participant per organization or institution was recruited, except for the government, where one person from national and another from district level were recruited. On the day of the interview, two participants appeared together with a colleague with whom they wanted to be jointly interviewed, resulting in *n* = 22 participants in 20 interviews. Although we aimed to include equal views of both male and female viewpoints in this study, male participants (*n* = 19) were heavily overrepresented due to their high presence in this field of work. Four participants were not of Sierra Leonean origin but were either West Africans (*n* = 2) or Europeans (*n* = 2) and had lived in the country for at least 3 years. After giving informed consent, the first author interviewed two participants via video call and met the remaining interviewees individually at their workplaces or at a mutually convenient location. Most interviewees were met in Freetown, the capital city of Sierra Leone, some in different parts of the country. All interviews were audio-recorded and minutes were recorded on non-audible events. Interviews lasted between 20 and 80 min (*M* = 53.35 min). Interviewees did not receive any incentives.

#### Local citizens

As we are interested in different experiences of inequalities of men and women in resource-low rural and urban communities, in which everyone is affected by certain conditions of poverty, our professional network served to identify eight communities in six different districts in Sierra Leone. A sample size of 16 per subgroup (female, male, rural, urban) determined the sample size. Upon arrival, the first author and the local research assistants introduced themselves to the contact person and the community chief to explain the purpose and procedures of the research project. Upon consent by the chief, the contact person was provided with a list of criteria (i.e., gender, fluency in the Krio language, primarily lower income and low education, not more than one person per household) and asked to suggest six or seven participants. To ensure that each of the required criteria were met, suggested participants were met in or at their house. After obtaining informed consent, each participant was interviewed by the local assistant of the same sex as the participant. A total of *n* = 56 citizens (50% women, 50% rural areas, 84% with fewer than 6 years of formal schooling) from four rural and four urban locations participated in the study. As 53% of residents in Sierra Leone [50] had not been registered at birth, exact knowledge of one’s age is not readily available and people tend to estimate their age or allocate themselves to an age range. For that reason, participants were assigned to three different age groups: young (estimated 18–29 years), middle (estimated 30–45 years) and old (estimated 46 years and above). With a life expectancy of 52 and 54 years for men and women respectively [51], the old-age threshold in Sierra Leone was set lower as it would have been done in high-income countries with higher life expectancies. Last, as the number of young- and middle-aged adults is considerably greater than older people in the total population [50], fewer old-aged people were recruited.

Interviews lasted between 15 and 35 min (*M* = 24.20 min). Each participant received a drink and a snack after the interview as compensation.

### Interview protocols

Semi-structured interview guides were developed to ensure that all relevant themes were discussed. Experts were asked about their perception of the status quo of handwashing practice in Sierra Leone; barriers and enablers to handwashing practice; differences between various social groups and their consequences for handwashing practice. For the local citizens, questions evolved around the following themes: sociodemographic data; current handwashing practice; perceived purpose, benefits and disadvantages of handwashing; barriers and potential enablers to regular handwashing; perceived differences between various social groups. Last, to learn about power structures and social change, local citizens were given a scenario and asked to imagine what would happen if the pump handle of their only community well broke. Community-owned, collectively used water wells with a pump handle are typically found in Sierra Leone and it is common that under frequent use, the handle of the pump breaks occasionally. In the absence of a tax system or public repair services, local citizens were asked to describe the events following the pump break.

### Data analysis

All interviews were transcribed verbatim. In those cases when data was collected in Krio, the translation to English and transcription process occurred simultaneously. The first author who is an experienced translator worked closely alongside her local assistants to allow for transcript accuracy and avoid the loss of meaning in the translation process. In the case of a metaphoric expression that does not have an equivalent in English, a note with an explanation of the meaning of the expression was made in the transcript. For anonymization purposes, all identifying data such as names of places, people, companies and organizations were removed and replaced by a description in square brackets, e.g., [village in the north of Sierra Leone].

In response to RQ 1 and 2, data were analyzed using thematic analysis [52], supported by the software MAXQDA [53]. A multistage analysis process was applied to analyze both data sets separately and then combined. To generate SEM determinants, deductive category application and inductive subcategory development guided the analysis [54]. An initial coding frame was built in line with the above-described themes of the two interview guides and new subcategories were generated as derived from the data [55]. In contrast, intersecting social identity dimensions were derived inductively from the empirical data. In the following step of axial coding, subcategories were reviewed, linked, aggregated and defined to ensure that they were mutually exclusive. All transcripts, coded segments and finalized coding frames were read once more carefully before comparing the two data sets. Coded transcripts were read again to determine whether themes and subcategories were suited to be matched. In addition, the interviews of local citizens were grouped by relevant sociodemographic variables (rural or urban location, male or female participant) and their subcategories were compared. In the last steps, the final coding frames of both data sets were compared.

To develop a typology of social positions with privilege and disadvantage in relation to handwashing (in response to RQ 3), our qualitative data was analyzed in three steps comparable to the procedure of quantitative cluster analysis [56]. Cluster analysis allows for the formation of groups of people (i.e., types) by allocating individual cases with shared similarities to groups. As a first step, the previously identified identity dimensions were used as binary variables and noted on file cards (e.g., variable ‘gender’ with the values ‘female’ and ‘male’). Each value was marked as associated with either more or less privilege. Coded segments of the transcripts were re-read to identify intersecting dimensions, which might increase experienced advantages or disadvantages or render them less important. Based on those intersecting social identity dimensions, social positions were formed by assigning these variables and their intersections to different groups. In the last step, these social positions were organized hierarchically on a continuum from most to least privilege for handwashing uptake. Table 1 gives an overview of the methodological approach.

**Table 1:** Overview of the methodological approach

Research interest	Data analysis	Sample
Multi-level determinants of handwashing (RQ 1)	Thematic analysis	Experts (*n* = 22) and local citizens (*n* = 56)
Intersecting social identity dimensions with handwashing (RQ 2)	Thematic analysis	
High and low social positions for handwashing (RQ 3)	Typology	

Interview data from the local citizens was coded by two coders individually, including the first author, and disagreements were discussed until consensus was reached. The expert interviews were coded by the first author. All coding frames and the typology were discussed thoroughly between the two authors.

Participants from the expert sample are referred to as experts and were assigned a number that reflects the order in which interviews were conducted, e.g., Expert, 1. Further information on the professional background of each expert can be found in Table 2. Each participant from the sample of local citizens was assigned a pseudonym that reflects his or her gender as well as a short code, describing the place of residence (R for rural or U for urban), gender (F or M) and a number for one of the eight locations of data collection, e.g., Kadiatu, RF7. Table 3 gives an overview of the sociodemographic data of local citizens in this study.

**Table 2:** Professional background of participants in the expert interviews (*n* = 22)

Code	Participant’s professional background	No. of participants	Sector
Expert (1)	International program manager	1	NGO
Expert (2)	Local community engagement officer	1	NGO
Experts (3.1 & 3.2)	Local nurse	2	Healthcare
Expert (4)	Local religious leader	1	Local leadership
Expert (5)	Local trainer of CHWs	1	NGO
Expert (6)	Local community engagement officer	1	NGO
Expert (7)	International journalist	1	Media
Expert (8)	Local paramount chief	1	Local leadership
Expert (9)	Local religious leader	1	Local leadership
Expert (10)	Local policymaker & program manager	1	NGO
Expert (11)	Local policymaker & program manager	1	NGO
Expert (12)	Local journalist	1	Media
Expert (13)	Local religious leader	1	Local leadership
Expert (14.1. & 14.2)	Local community engagement officer	2	NGO
Expert (15)	Local program manager	1	NGO
Expert (16)	Local nurse	1	Healthcare
Expert (17)	Local government official, national level	1	Government
Expert (18)	International media producer	1	Media, NGO
Expert (19	Local government official, district level	1	Government
Expert (20)	Local media producer	1	Media, NGO

**Table 3:** Demographics of local citizens (*n* = 56)

Characteristics	***n***
Gender	
Female	28
Male	28
Location	
Rural	28
Urban	28
Age group	
Young	24
Middle	20
Old	11
Missing	1
Education	
No formal education	31
Primary school	14
Secondary school	10
College/University	1

### Positionality and reflexivity

The first author had resided in Sierra Leone for 2.5 years over the course of 5 years and was acquainted with culturally appropriate expressions and behavior prior to this study. Nevertheless, as a White, educated, unmarried woman from a Western country, she remained an outsider, which has both advantages and disadvantages. The local gender norms were problematic when accessing and maintaining control in three expert interviews with high-ranking male officials: After postponing the interview appointments numerous times, these participants dominated the conversation by talking continuously, hardly allowing for interposed questions. However, as they were elaborate and their lengthy responses tended to answer multiple questions to be asked in one, all three interviews were kept in the sample. In contrast, encounters with local citizens went smooth.

### Ethics, consent and permission

This study gained approval from both the Sierra Leone Ethics and Scientific Review Committee and the ethical committee of the University of Erfurt, Germany. All study participants were informed about the project goals, topic, type of questions to be asked and their right to decline participation or to withdraw from the study at any time. Participants were asked if they had any questions prior to giving informed consent. In the case of illiterate participants, an impartial witness was present while the study purpose and procedures were explained. The participant thumbprinted the informed consent form in the presence of the witness who then also signed the consent form. All our procedures have been performed in accordance with the ethical standards as laid down in the 1964 Declaration of Helsinki and its later amendments.

## Results

### Determinants within the socioecological model (RQ 1)

Analysis of the interview data with the two different samples yielded various determinants of handwashing within each of the three levels in the SEM (see Supplementary Table 1 with citations for each theme).

#### Individual level

At the individual level, the great majority of local citizens expected positive outcomes from handwashing, e.g., in the area of disease prevention:

If you wash your hands, there are some sicknesses that will not affect you. (Saa, RM6)

While life-threatening diseases such as cholera and Ebola were mentioned the most, diarrhoea and other less-lethal conditions were stated considerably less. Moreover, being physically and spiritually clean, the latter cited mostly by Muslims, were frequently mentioned purposes of handwashing. However, it became apparent that local citizens’ knowledge was incomplete regarding critical moments of hand hygiene, e.g., before food and after faecal contact, what the purpose of soap was and how handwashing was linked to infection prevention and better health. Experts explained this by a lack of formal education and resulting knowledge gaps in biomedical processes:

How many people can realize that, okay, when my kids wash their hands frequently, they are not having any faecal-related infections? I know that if I wash my hands after using the restroom or something, it will prevent some diarrheal diseases. But how many people do know that at local community level? For our people what they can’t measure, what they can’t see, there is no importance for it. (Expert, 15)

Moreover, it was pointed out that diarrhea might even be aspired in little children as the following quote illustrates:

Because there is a cultural belief to diarrhea for children who are trying to walk and everything. For me, when I was growing up and when I had my first child, my mother would say ‘This is a sign that the child will go to the next stage to start walking.’ (Expert, 5)

While experts described how misconceptions and lacking knowledge altered risk perceptions about the severity of diseases such as diarrhea and preventive measures, various local citizens contradicted these expert views as they expressed interest in increasing their handwashing practice but felt unable to do so due to missing resources (e.g., a sustainable water source and soap), perceived social support and a lack of habit:

There is extreme poverty in Sierra Leone and as such, not everyone can afford to buy soap to wash hands even though they’d have wished to wash their hands with soap. (Momoh, RM8)

I am stressed for time nowadays and not able to wash my hands with soap and water because I get back home from work very tired and I forget. (Hassan, 5UM)

#### Social level

Participants agreed that handwashing for hygiene purposes was not ritualized and a part of common social protocols in Sierra Leone. Moreover, asking guests to wash their hands was associated with social expectations of having to serve food as some local citizens stated:

Some were saying that you just have to wash your hands when there is food because some people were thinking, at every point I wash my hands, there will be food. So, when you asked them [visitors] to wash their hands and they are not seeing food, they ask, ‘What is the reason I have to wash my hands? I am not seeing food, but I am washing my hands.’ So, because of these people who were provoking others, some people were not washing their hands again. When you ask these people to wash their hands, he [the guest] will tell you that you will have to cook for him before leaving. (Foday, RM8)

However, handwashing is also positively connotated on a social level. Islamic ablutions, referring to ritual handwashing without soap for spiritual cleansing, are consistently practiced five times a day among large proportions of the population yet without spillover effects to handwashing with soap for disease prevention:

It is interesting that when Muslims go to pray, they thoroughly wash their hands. So, from that point of view, the hygiene aspect is kept very tight. But the everyday practice after the toilet, that's interesting, it gets neglected. (Expert, 1)

Together with rather negative social norms of handwashing beyond religious cleansing, a lack of role models was frequently mentioned by experts and local citizens. Above all others, medical workers were described as the most credible role models and promoters of handwashing yet are absent in the daily lives of people.

#### Structural level

Both experts and local citizens agreed on the structural resources necessary for handwashing. On the one hand, it required year-round, sufficient water supply, access to soap and a handwashing station. The former was said to be a great challenge in rural areas as well as during the dry season when water resources became scarce in urban and rural areas.

There needs to be a ready source of water and soap to enable me wash my hands. But not everyone in this country has it. (Hawa, UF3)

On the other hand, to promote handwashing and make it a regular topic in everyday conversation, a good communication infrastructure, especially audio and audio-visual channels amid high illiteracy rates, were considered key components for handwashing practice.

### Social identity dimensions intersecting with handwashing practice (RQ 2)

**Fig. 1:**
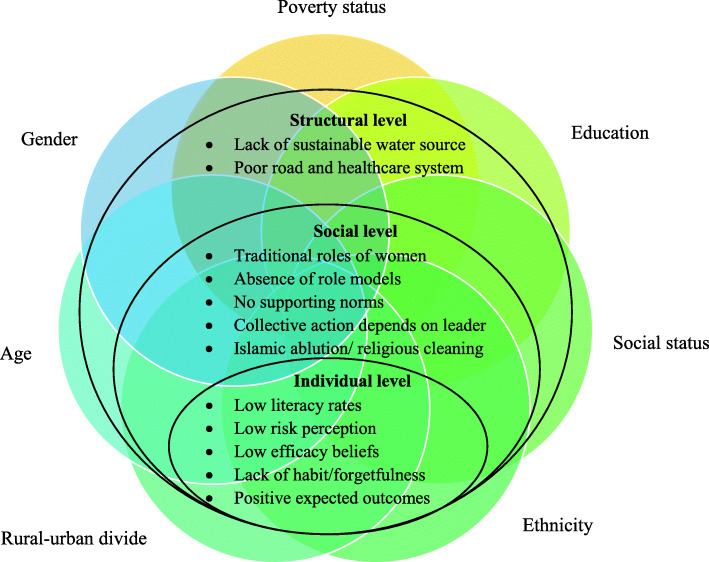
Seven intersecting dimensions of handwashing behavior in an SEM

Our data indicates that seven identity dimensions intersect with handwashing practice. The seven identified dimensions are briefly described as singular inequalities with an illustrative quotation for each dimension. Figure 1 represents these intersecting dimensions on the three levels of an SEM.

Additionally, the intersection of several dimensions is illustrated through the profiles of the most and least disadvantaged individuals under RQ 3.

#### Poverty status: narrow margin of options amid lacking resources

Participants from both samples described poverty as the greatest obstacle to handwashing practice. In some cases, the absence of the crucial resource water impaired handwashing efforts altogether. Additionally, experts described that as the effects of handwashing were not immediately visible, more urgent needs like food, were prioritized over purchases of soap:

When you go to the slum communities, you will know that they can't even afford to buy soap, because they need to eat first. Even if you say, okay you can use ashes from when you cooked your food – some don't even have ashes, because they don't even prepare food for the day. (Expert, 5)

#### Gender: women’s traditional work and lack of autonomy

Participants described the traditional work of women evolving around food preparation, childcare, caring for sick and older people – thus key moments of handwashing. While experts emphasised women’s handwashing was key to the health of the whole family, participants from both samples described an overall lack of autonomy (i.e., power to decide on own actions) women experienced. Having little say in financial decisions and staying in the background during community meetings, women experience few opportunities to buy soap and influence the increase of handwashing in their home as the following account suggests:

As a woman, there is a limitation as to what I can do, even though if I believe, even if I accept, when the people around me, you know, don’t believe in what I say, I will find it difficult. (Expert, 10)

#### Rural-urban divide: poor rural infrastructure with tight community cohesion

Participants reported how the differences in the infrastructure affected information access and handwashing practice of rural residents in multiple ways. Rural areas were frequently described to be disproportionately affected by a poor infrastructure with implications for people’s access to a sustainable source of water, healthcare and health information. While experts acknowledged that in some cases, there was no need to promote handwashing if there was no water, they also pointed out the tight community cohesion and available social capital in rural communities as an advantage over urban areas:

Everyone is a member of one group at least: the youth group, the women’s group, the development group. So, we help each other out. (Foday, RM8)

Especially those rural communities are more without water… So when water is not available and you have to pay for it and there is no money, how do you practice the behavior? You cannot. So that is a challenge more than in the cities. (Expert, 5)

#### Age: respect for the old and the continuation of traditions

Another obstacle to behavior change was said to be the age gradient between older and younger people. Older people are influential family members and respected for their knowledge, life experience and contribution to society. They uphold traditions and might oppose new behaviors, especially if they diverge from the status quo. Various experts described health programs in which they had targeted only younger people such as school children or youth, yet with limited success due to their lack of influence at home. Instead, the inclusion of both older and younger generations could contribute to changing existing social norms as the following quote illustrates:

We had a role play with a grandfather and his grandson. So, there was this grandfather who because of his knowledge thinks, you know, everything he does is right. Then there was the grandson correcting the grandfather and because of the drama, because of the fun, people were laughing, but in the process, they were also benefiting. (Expert, 20)

#### Social status: power of local leaders

Experts described a strongly hierarchically organized society in Sierra Leone with community leaders, such as chiefs, religious and tribal leaders, at the top of the social hierarchy who experience executive power and status. Although women are not excluded from taking certain leadership positions, e.g., a few experts mentioned a women’s leader, most positions appear to be inhabited by men. Especially experts described the ambivalence and potential dual influence of these leaders: On the one hand, local leaders were described as “custodians of traditions” (Expert, 12) that can prevent the uptake of health behaviors, especially if they are against local traditions; on the other hand, they can lead and enforce the change:

When the leaders take the decision, you should follow it. When they put by-laws, and you don’t do it, you will be punished…. Sometimes even when they are not doing the right things, people don’t have an alternative. (Expert, 6)

The leaders in the community will call a meeting and discuss issues before decisions are taken. The decision is later communicated to the entire community. (Tamba, RM6).

#### Education: a lack of schooling and restricted access to information

There was overwhelming agreement that a lack of formal education was disadvantageous to handwashing practice. While some literate local citizens had learned about handwashing and disease prevention during their years of schooling, the majority of local citizens with no or few years of primary school had only heard about handwashing through health programs. Moreover, illiteracy as the result of low educational attainment had consequences for people’s access to information:

Some people are educated and some people are not educated. So when they were coming, they often target more for people that are educated. The people who will understand are the people that are educated, but not everybody went to school. (Hassan, UM5)

In Sierra Leone, school attendance is compulsory, but we still have an adult illiteracy rate estimated at over 50 %. In our work, we see every day that education is very, very crucial in how people talk about health and where they get information about health. (Expert, 1)

#### Ethnicity: predominance of some languages to the neglect of others

The multiple tribes of Sierra Leone with each their own tribal language were described as a well-known obstacle to health promotion programs. Participants from both samples described it as disadvantageous to having been raised in only one tribal language. This way, being monolingual limits a person’s options to understand health messages and communicate with others outside one’s immediate social circle. While there is a concentration on Krio, the local language mostly spoken in the capital area where also most media organizations and NGOs are situated, experts acknowledged that multiple tribal languages often had to be neglected because of the high costs of translation:

When we do radio, we literally have to get a new group of actors with those language skills. We take the same scripts and we re-record it…. Where we can, we work in local languages, but it is always a trade-off in the context of Sierra Leone with a limited amount of funding. (Expert, 18)

### Most and least disadvantaged social positions regarding handwashing practice (RQ 3)

Developing typologies of the data revealed disadvantages and privileges resulting from multiple intersecting social identities that certain social positions hold. While the dimensions poverty status and education were shown to affect handwashing behavior of all groups under research, the effects of these disadvantageous dimensions vary considerably depending on other social identities of the individual.

The following outline characterizes the two extremes in this social spectrum and how the combined social identity dimensions shape each person’s access to information about handwashing as well as the practice itself. The most disadvantaged social position in this respect consists of a rural, low-educated, poor, young wife raised in one tribal language. In comparison, an older man in a leadership position independent of his education or place of residence is most privileged to information access and washing hands with soap.

#### Most disadvantaged: rural, low-educated, poor, young wife raised in one tribal language

Participants of both samples described the interplay of multiple social inequalities and structural disadvantages of a rural, low-educated, poor young wife who speaks only one tribal language regarding handwashing practice. This section begins with the dimension rural-urban divide as the social and structural conditions of a village have shown to aggravate several other dimensions for rural women who share other characteristics with their urban counterparts. First, there was agreement that there are crucial differences between urban and rural women regarding their access to health information. One aspect is the communication infrastructure:

This is an urban setting, we can provide information: We have electricity, we have television sets, we have mobile phones. We are not that much vulnerable. (Expert, 12)

In contrast, villages in Sierra Leone are usually not connected to the electricity grid which drastically reduces the number of available communication channels. Battery-powered radios were said to be the most frequently used communication device, but strict gender hierarchies give rural women less decision-making power over their use:

The father will be in charge [of the radio] and, two, if the battery is not there, the father will buy the battery and he will restrict the children or the women not to listen. (Expert, 12)

Second, as rural, low-educated women are likely to be fluent in a tribal language other than Krio, radio programs that meet their linguistic needs are few:

The question is how many of them [the women] can understand because the language being used most times is Krio, only few radio stations can really broadcast in the local languages full time… We assume that everybody can understand Krio and for me that is not correct. We need to talk in our own languages that they [women] will understand better. (Expert, 12)

Third, healthcare centers are within close proximity in the cities and easily reachable by public transport on good roads, participants from both samples agreed that seeking healthcare for themselves and their children was easy for urban women. This way, young, urban women tend to be regularly in touch with the healthcare system and receive personalized health information from medical workers. Their rural counterparts are likely to live at some distance from the nearest healthcare institutions with non-existent or irregular public transport and poor roads. Due to these lapses in the infrastructure, rural women are considerably less exposed to face-to-face encounters with medical personnel:

We are having lots of unreachable communities due to road network. So even for the free healthcare, we have a lot of communities that do not benefit from the free healthcare as they are just not reachable. And these people who do the health education, when they do have health promotion, they target those easy to reach places. So, for people living in those hard-to-reach communities, they hardly benefit from these health facilities. (Expert, 20)

In addition, as the work of rural women is often bound to their homes and agricultural duties, they experience few opportunities to seek for information independently and rely on the information their husbands or others bring back:

The men [get more information] because they go out for work and they walk around a lot. Women stay at home while the men go out. (Fatima, RF4)

Men walk around a lot, they even go to the junction to get the news. We rely on them to bring us the information. (Humu, RF7)

In contrast, agricultural work is largely uncommon in cities. The majority of female city dwellers in our sample often work in petty trading and take daily trips to local markets where they meet many different people and can inquire information independently. For the few available communication channels in rural locations, experts and local citizens agreed that visiting villages was the safest way to ensure both men and women received first-hand health messages. However, even in a community-based setting, various experts pointed out difficulties in giving equal access to information as “cultural beliefs put women not as high as compared to men” (Expert, 11). Women would more often be absent or not participate as this hygiene trainer explained:

There are places where women do not sit where men are in these remote communities. Up till now, there are some of these places where women are not allowed to participate fully, you know…. They don’t talk too much because they might not be allowed in such a forum next time, you know. But they are suffering the most and for me, the women they are almost always dealing with the problems. (Expert, 2)

While large community-based meetings are overall less common in the cities, being informed about health issues gave women more confidence even to speak in public forums or in their homes as a program manager pointed out:

In the cities, because of their access to information, women gain a better understanding about the world surrounding them and what is good for them. This makes them more self-confident and they can even convince their husband to change. (Expert, 1)

There was agreement that receiving health information and being knowledgeable about handwashing was not equivalent to being able to put it into practice. Poverty, gender and age divides were described as intersecting identities that affected handwashing practice. Here again, living in a village exacerbates these dimensions. For instance, while urban dwellers occasionally experienced water shortages, obtaining sufficient water was described as an omnipresent issue in rural communities as wells dry out during the dry season and available water sources are often at a distance:

If there is enough water, handwashing is easy, but when the water is far, it is very hard and we cannot do it all the time. (Kumba, RF4)

Moreover, as men are in charge of allocating money, yet rather unaware of the duties of women, soap purchases were constrained by gender inequalities. In rural locations, men were said to be almost exclusively responsible for financial decisions and, thus, whether soap for handwashing was bought or not. In urban settings, there were different views over women’s influence in financial decisions. While some women with an own income determined its usage, others said their husbands often continued to have the final say:

I am man and woman in one person: I am the breadwinner in my family and I do the housework. My job is house cleaning and I have done it for the same people for many years, but my husband only works occasionally…. When I sometimes say, ‘I want to buy this’, he will say ‘Let us wait until tomorrow.^1^’ (Yainkain, UF2)

Apart from men, young wives in cities and villages are hierarchically subjugated to older women in their household. As they often live together with other members of their husbands’ family aunts or their mother-in-law can further limit health behaviors if they disagree:

You have mothers who want to breastfeed, but maybe their mothers-in-law don’t want them to breastfeed. Or they want to go to the clinic to deliver, but their husbands will say, ‘No, you can’t deliver in the clinic, you deliver at home.’ (Expert, 10)

She [mother-in-law] will tell the woman, ‘I'm forty years old and you know, I've done it all my life and nothing has happened to me. I mean look at me, I look healthy, I'm very strong. You cannot just come and change my mind.’ (Expert, 14.1)

Taken together, the compounding social identity dimensions of a rural, low-educated, poor, young wife who is fluent only in one tribal language provide substantial obstacles to her information access and uptake of handwashing practice. Analysis of our data has shown that each dimension adds limitations and disadvantages to her ability to get informed about health matters and to adopt the practice. While both urban and rural women experience restraints by poverty, low education, gender and age, the structural and social conditions of a village aggravate these factors and greatly limit a rural woman’s ability to perform handwashing. In contrast, urban women with the combined effects of access to a better infrastructure (communication, healthcare and water), some financial income and increasing health knowledge are more privileged to take up handwashing.

#### Most privileged: older man in a leadership position

As described elsewhere, the dimensions low education and poverty equally affect members of the local populations and are, thus, not further described in this section. Moreover, experienced disadvantages and, conversely, privileges based on gender, age, rural-urban divide and ethnicity were explained in detail in characterizing the young wife. While not every old man is a leader, our data indicates that being in a leadership position is often equivalent to being a man with a certain age and life experience. Therefore, this section focuses on the social status associated with a leadership position and the resulting opportunities for handwashing practice. On the positive side, leaders are considered both role models whom others would follow voluntarily as well as equipped with executive power that allow them to enforce behaviors. There was unanimous agreement that the power of local leaders goes even beyond what the government could achieve as member of the Ministry of Health described:

Communities have their structures and whatever has been agreed upon by the leadership of the community and anyone violates it, there will be a penalty. The Ministry of Health is not able to institute penalty, but if the community leaders agree, they have their structures and they can make sure people obey according to their discipline structure. (Expert, 11)

Furthermore, due to other privileges such as good access to information and being connected to people outside their community, they can serve as information source to their communities as well as feeding back community views to those implementing health programs. However, experts also acknowledged that community leaders stand for certain worldviews and traditions. As a result, leaders could use their influence in either way and also thwart the implementation of health programs:

I think the authorities need to look at this, how to control [these leaders] though I know you can’t just control them… We have seen this at play with regards to vaccination. The Muslims in particular are very opposed to vaccination, but mostly based around religion. The problem is to overcome this. (Expert, 7)

Due to their dual influence over communities, several experts were of the opinion that collaboration and the involvement of leaders was indispensable to achieve positive outcomes:

If they are able to convince people to not go to the facilities, then it means they have power. It’s either they are a part of the problem or they are part of the solution. (Expert, 10)

Sometimes it [behavior change] is best achieved by targeting a group of people directly, sometimes it’s best to reach them by targeting another group of people who might, for example, have too much power in the equation. (Expert, 18)

It is worth mentioning that leaders were considered more influential in rural settings with tight, relatively stable social networks than in urban settings with loosening social cohesion.

To summarize, the social position of an old, male leader allows him to access information relatively independently or even enjoy exclusive access by being targeted by government authorities and other implementers to spread information or execute the implementation of health programs. Positioned at the top of the social hierarchy, leaders can maintain the status quo or empower disadvantaged individuals by impacting powerful others, e.g., encourage husbands to allocate money for their wives’ soap purchases.

## Discussion

While the importance of handwashing for infection prevention had long been established and addressed by numerous frameworks, the COVID-19 pandemic has given handwashing global attention and demonstrated how this crisis exacerbates existing inequalities associated with handwashing [57]. Moreover, handwashing frameworks have been criticized for their bias towards certain sets of determinants (e.g., individual-level or structural determinants) because they risk missing its complexity [17]. In contrast, socioecological frameworks allow for the conceptualization of multi-level determinants of handwashing and have shown the high relevance of social- and structural-level factors in addition to individual factors [58, 59]. However, they do not explicitly take into account power gradients and differing degrees of vulnerability between groups in a shared social setting. The consequences of non-uniformly experienced social inequalities in relation to handwashing and hygiene as demonstrated during the pandemic [9, 60] can be brought into focus by an intersectional approach. With a blended socioecological and intersectional approach, our study thus responds to the necessity to explore the embeddedness of handwashing practice in its social and structural environment and account for inequalities between groups with distinct social identities. To our knowledge, this combined approach has never been used for handwashing and provides a complex perspective on its practice in a LIC. While our study employed only qualitative methods, a combination of qualitative and quantitative methods could further enhance the data quality.

The multi-level exploration of handwashing determinants has shown how individual handwashing practice is shaped by factors in the social and structural environment. In addition, by establishing seven distinguishing social identity dimensions, we could specify the interconnectedness of multi-level determinants and identity dimensions and demonstrate that individuals within the same social setting experience differing degrees of privilege and disadvantage to enact handwashing. By looking at social stratifications within this group that all experience certain degrees of deprivations, our study adds new facets to conceptualizing barriers and enablers to handwashing in a socioeconomically similar yet still heterogeneous population group. While other studies focused only on one social identity dimension, such as gender (e.g., [61]), our study approach did not have a predefined focus on one dimension. Although we did not include the whole variance that would have been possible for the dimensions, we included those parameters that are most relevant for interventions.

By applying an intersectional approach, we developed a nuanced understanding of intersecting social dimensions and the heterogeneity within social groups. This became most apparent when comparing young wives in urban and rural areas whose social characteristics are very similar yet the associated privileges and disadvantages of their location of residence exposed them to distinct opportunities and challenges for handwashing practice. This way, the improved infrastructure of a city allowed the urban woman to learn about hygiene and other health topics, access a better infrastructure and earn some money which could then counterbalance some of the disadvantages she shared with her rural counterpart (e.g., lack of education and influence in her household). Taking a different example, the age gradient as a distinctive factor between two otherwise similar rural women allowed the older female to impose considerable influence over a younger one with implications for the enactment of handwashing of the younger woman.

### Theoretical and methodical implications

With regard to theoretical implications, our study provides a holistic analytical framework for the identification of determinants on multiple levels and the intersection of social identity dimensions in a given environment. Thus, socially produced inequalities and power imbalances that contribute to a lack of health behavior are not neglected or ignored [33, 62], but rather identified from a solution-focused instead of a problem-focused perspective. We sought to portray the power gradients between different social groups in a shared social system and the disproportionate barriers some individuals experience. Consequently, our analysis of intersecting privileges and disadvantages also resulted in revealing important powerholders, who can serve as agents of change to empower those most disadvantaged [63]. Employing an intersectional perspective allows to identify different degrees of experienced disadvantages and demonstrate the striking differences of accumulated social identities and their impact on a person’s opportunities to enact a health behavior. As done in the early years of intersectionality theory [28], our analysis provides a spectrum with nuanced views on different social groups who are all disadvantaged in at least two dimensions (poverty, education). Accumulating intersections of further disadvantages render some individuals subject to increased vulnerability and marginalization. This way, our findings resonate with other studies that increasing disadvantages lead to an intensified vulnerability to enact health behaviors [39, 64, 65].

This holistic theoretical approach is also reflected in the rather comprehensive methodical approach which mapped the different levels of determinants and social identity dimensions by relying on two different samples with multiple social differences.

### Implications for policy and practice

Regarding the different level of determinants, our analysis can guide different intervention approaches. While the absence of a sustainable source of water, for instance, calls for a technical intervention, the identification of a lack of health knowledge requires health communication interventions to increase hygiene literacy. This also means that as long as sustainable water and soap supply are missing, the promotion of handwashing with soap will be fruitless. By taking a holistic and integrative perspective on handwashing behavior, we could identify starting points within the complexities of multi-level determinants and intersecting social identities. This way, our study of power gradients can serve as a formative study to a participatory, community-based project where all social groups are involved throughout different phases of the project. While those with less power are empowered and partake in decisions about handwashing, those with more power can be addressed to support them with their resources.

Our analysis also highlights the role of influential, rather resourceful leaders who can be employed as opinion leaders or agents of change [63]. Identifying and employing influential individuals such as local leaders has shown to positively influence the behaviors of different social groups in public health emergencies [66, 67] and non-emergency settings [68, 69]. They could contribute to empower disadvantaged but relevant individuals for handwashing, e.g., increase community-based health communication activities (most recommended in villages) and thereby allow rural, young wives to increase their health literacy. At the community level, leaders could contribute to setting community or social norms by creating and monitoring binding rules to increase the uptake of handwashing. Their promotion, instead of messages by outsiders, may allow for the formation of new, positive social norms connected to positive emotions of being clean (spiritually and physically). On the structural level, leaders could lead the development of locally apt solutions, e.g., use local materials for a handwashing station instead of buying some, create nudges to not forget handwashing, share soap or create a community fund to provide soap to those in need. As leaders, they can also make interventions participatory and allow communities to ‘own the problem’ instead of being dependent on unpredictable supplies from outside the community. However, influencing leaders in processes of change might require a different strategy, especially if the behavioral change threatens their power or requires a diversion from traditional practices.

### Limitations and further research

This study has certain limitations. Research was carried out in Sierra Leone and our findings reflect a snapshot of the social and structural conditions of handwashing in this country. Although we sought to reflect the diversity of different social groups, the seven identified dimensions might not be applicable in a different context or even within Sierra Leone regarding a different behavior. By applying a similar approach in a different LIC, future studies can enhance our insights into power relations and social and structural disadvantages in resource-low environments and, hence, contribute to validating the analytical approach of a combined socioecological and intersectional approach. When this holistic approach is applied to another health topic, other settings or different health systems, it can ensure to identify suitable interventional strategies. While our study makes several suggestions on how this combined socioecological and intersectional approach can inform interventions and processes of change, further research is required to conceptualize it into a measurable approach to be applied in interventions. A longitudinal study design would be particularly insightful to measure not only behavior change and its effects on health outcomes, but potential shifts in power relations over time. There are also methodological limitations worth mentioning. There may have been selection bias as local citizens were recruited based on suggestions by their respective community chiefs. This sampling technique is typical in the context of Sierra Leone and by using a set of criteria, we aimed at mitigating the effects of not having direct influence over the sampling process. Moreover, we concentrated on adults in this research project and the views of minors are not reflected in our findings. Power imbalances are also reflected in the length of statements by the interview partners. Thus, participants of higher SES (i.e., experts) have given more insights into their opinions than people with lower SES.

A social desirability bias may have affected our data as participants framed their responses differently in the face-to-face interviews. In addition, the positionality of the main researcher and her different cultural and socioeconomic background may have inhibited some participants from how they expressed their views.

## Conclusion

Despite its immense contribution to public health, handwashing with soap is least practiced in LICs. While studies have shown how handwashing behavior is shaped by a multitude of contextual influences, their interactions have often been neglected in recent studies. Against this gap in the literature, our study explored how handwashing is embedded in the social and structural environment of a LIC and how distinct social identities in a shared setting frame how inequalities and privileges are experienced. By blending a socioecological and intersectional approach, this study provided a unique perspective on handwashing in a LIC and allowed for the identification of fruitful starting points and important agents of change for handwashing promotion.

## Supplementary Information

**Additional file 1: Supplementary Table 1.** Description of determinants of handwashing within the SEM.

## Data Availability

The datasets used and/or analyzed during the current study are available from the corresponding author on reasonable request.

## Abbreviations

FOAM: Focus, Opportunity, Ability and Motivation
HWWS: Handwashing with soap and water
LIC: Low-income country
NGO: Non-governmental organization
SEM: Socioecological model
SES: Socioeconomic status

## Footnotes

1 “Tomorrow” is an often-used expression in Krio to refer to an unknown point in time in the future or never.
