# Correction to: Embracing challenging complexity: exploring handwashing behavior from a combined socioecological and intersectional perspective in Sierra Leone

**DOI:** 10.1186/s12889-021-12291-6

**Published:** 2021-12-13

**Authors:** Hanna Luetke Lanfer, Doreen Reifegerste

**Affiliations:** School of Public Health, Bielefeld University, Universitaetsstrasse 25, 33615 Bielefeld, Germany

**Correction to: BMC Public Health (2021) 21:1857**

**https://doi.org/10.1186/s12889-021-11923-1**

In this article [1] ref. 47 was missing and the first sentence of the Methods sections should be as shown below. The references and their in-text citation after the updated reference 47 have been renumbered. The original article has been updated.

**Methods**

**Study design and setting**

This research is part of a larger study to promote handwashing in a low-income environment [47].
