# Supplementary material for: Embracing challenging complexity: exploring handwashing behavior from a combined socioecological and intersectional perspective in Sierra Leone
Source: BMC Public Health. 2021 Oct 14;21:1857. doi: 10.1186/s12889-021-11923-1 (PMC8515313; doi:10.1186/s12889-021-11923-1)
Supplement: Supplementary file 1 — Additional file 1: Supplementary Table 1. Description of determinants of handwashing within the SEM. [file 12889_2021_11923_MOESM1_ESM.docx]

*Supplementary Table 1: Description of determinants of handwashing within the SEM*

| **Level** | **Determinant** | **Example** | **Sample citation** |
| --- | --- | --- | --- |
| Individual | Knowledge | Partial knowledge about germs and how handwashing during key moments can prevent sicknesses | How many people can realize that, okay, when my kids wash their hands frequently, they are not having any faecal-related infections? I know that if I wash my hands after using the restroom or something, it will prevent some diarrheal diseases. But how many people do know that at local community level? For our people what they can’t measure, what they can’t see, there is no importance for it. (Expert, 15) |
|  |  | Misconceptions about diarrhea in children | Because there is a cultural belief to diarrhea for children who are trying to walk and everything. For me, when I was growing up and when I had my first child, my mother would say ‘This is a sign that the child will go to the next stage to start walking.’ (Expert, 5) |
|  |  | Perceived insufficient information due to illiteracy, not owning a radio or phone, living in a remote area or being a woman | Men walk around a lot, they even go to the junction to get the news. We rely on them to bring us the information. (Humu, RF7) |
|  | Risk perception | Perceived susceptibility towards diseases such as Ebola, cholera and diarrhoea | If you wash your hands, there are some sicknesses that they will not affect you. (Saa, RM6) |
|  |  | Not perceiving the severity of diarrhoea as a serious health threat or perceiving it as normal in children and adults | While during the Ebola epidemic, people washed their hands because they face a lethal disease, this is not the case for diarrhea. The consequences of diarrhea are not seen as that serious. (Expert, 1) |
|  | Positive expected outcomes | Feeling positive about being clean and having a good body scent | The desire to keep a clean and decent appearance motivates me to wash my hands. (Kandeh, RM8) |
|  | Social support | Perceiving lack of social support or expectations of handwashing | People are not used to it because after the Ebola, people started mocking others, saying for how long are we going to wash our hands? That was why we found it very difficult to wash our hands. (Brima, RM6) |
|  | Control beliefs  Social norms | Feeling unable to practice regular handwashing due to a lack of resources (e.g., time, sufficient water or money to purchase soap) | If there is enough water, handwashing is easy, but when the water is far, it is very hard and we cannot do it all the time. (Kumba, RF4) |
|  |  | Perceived external expectations to practice handwashing and, thus expecting these bodies (e.g., medical workers, the government or NGOs) to supply all necessary resources | We are asking Papa Government, because they can help us to have better health. So, we wait for them to let them help and supply us. (Fatima, RF4)  If the government wants us [to wash our hands], we want the government and the NGOs to bring it back. (Sheku, RM8) |
|  | Habit | Lacking habit and forgetting about handwashing makes it difficult to remember | I am stressed for time nowadays and not able to wash my hands with soap and water because I get back home from work very tired and I forget. (Hassan, 5UM) |
|  | Conflicting goals | Making trade-offs between immediate (e.g., food for today) and long-term needs (e.g., soap to reduce likelihood of diarrhea) in the face of financial constraints | When you go to the slum communities, you will know that they can't even afford to buy soap, because they need to eat first. (Expert, 5) |
| Social | Religious norms | Washing hands without soap to perform the Islamic ablution is assumed to be equal to handwashing for soap for health purposes | It is interesting that when Muslims go to pray, they thoroughly wash their hands. So, from that point of view, the hygiene aspect is kept very tight. But the everyday practice after the toilet, that's interesting, it gets neglected. (Expert, 1)  Because we are Muslims, we already wash our hands. (Alpha, UM1) |
|  | Negative social expectations | Asking guests to wash their hands upon arrival required food serving by the host, causing unwillingness to ask guests to wash their hands | Some were saying that you just have to wash your hands when there is food because some people were thinking, at every point I wash my hands, there will be food. So, when you asked them [visitors] to wash their hands and they are not seeing food, they ask, ‘What is the reason I have to wash my hands? I am not seeing food, but I am washing my hands.’ So, because of these people who were provoking others, some people were not washing their hands again. When you ask these people to wash their hands, he [the guest] will tell you that you will have to cook for him before leaving. (Foday, RM8) |
|  | Gender norms | Traditional duties of women (e.g., food preparation, caring for the sick and children) are associated with her family’s health | I am a suckling mother, any time I want to touch my child, I should wash my hands, so that I and my child will feel healthy. (Amie, RF4) |
|  | Role models | Healthcare workers are perceived as role models and supporters of handwashing yet are not present in participant’s daily lives | It was the health workers who visited our communities that sensitized us on hand washing. (Jeneba, UF3) |
|  | Health promotion | Preferring continuous provision of information in oral formats, preferably community-based | They should come to the grassroot level, to the people and talk to them, so that they can know what they should do (Usman, RM6)  The government should go to the radio and talk so that people will understand what is going on. But they should not leave it just like that … because education is one thing and understanding is yet another thing. I think the government is in the best position to send some people to different areas to sensitize people so that people will understand it better. (Alpha, UM1) |
|  | Social capital | Strong social bonds especially in rural areas, but respected leaders are required to initiate collective action | Everyone is a member of one group at least: the youth group, the women’s group, the development group. So, we help each other out. (Foday, RM8) |
| Structural | Communication infrastructure | No or interrupted flow of electricity prevents attendance of radio or TV health programs | Why I am still insisting on the rural communities is because this is an urban setting, we can provide information: We have electricity, we have television sets, we have mobile phones. We are not that much vulnerable. (Expert, 12) |
|  | Water source | A year-round, sustainable water source is not a given and hampers handwashing, especially in the dry season | There needs to be ready source of water and soap to enable me wash my hands. But not everyone in this country has it. (Hawa, UF3)  If you say ‘Wash your hands with soap and water’, then you expect that you should always have water and soap. In most communities, water is scarce, even water to drink. So, they cannot afford, you know, to just wash their hands, when water is limited. (Expert, 10) |
